# High sulfur-containing carbon polysulfide polymer as a novel cathode material for lithium-sulfur battery

**DOI:** 10.1038/s41598-017-11922-6

**Published:** 2017-09-12

**Authors:** Yiyong Zhang, Yueying Peng, Yunhui Wang, Jiyang Li, He Li, Jing Zeng, Jing Wang, Bing Joe Hwang, Jinbao Zhao

**Affiliations:** 1 State Key Laboratory of Physical Chemistry of Solid Surfaces, State-Province Joint Engineering Laboratory of Power Source Technology for New Energy Vehicle, Collaborative Innovation Center of Chemistry for Energy Materials, College of Chemistry and Chemical Engineering, Xiamen University, Xiamen, 361005 P.R. China; 2 NanoElectrochemistry Laboratory, Department of Chemical Engineering, National Taiwan University of Science and Technology, Taipei, 106 Taiwan

## Abstract

The lithium-sulfur battery, which offers a high energy density and is environmental friendly, is a promising next generation of rechargeable energy storage system. However, despite these attractive attributes, the commercialization of lithium-sulfur battery is primarily hindered by the parasitic reactions between the Li metal anode and dissolved polysulfide species from the cathode during the cycling process. Herein, we synthesize the sulfur-rich carbon polysulfide polymer and demonstrate that it is a promising cathode material for high performance lithium-sulfur battery. The electrochemical studies reveal that the carbon polysulfide polymer exhibits superb reversibility and cycle stability. This is due to that the well-designed structure of the carbon polysulfide polymer has several advantages, especially, the strong chemical interaction between sulfur and the carbon framework (C-S bonds) inhibits the shuttle effect and the π electrons of the carbon polysulfide compound enhance the transfer of electrons and Li^+^. Furthermore, as-prepared carbon polysulfide polymer-graphene hybrid cathode achieves outstanding cycle stability and relatively high capacity. This work highlights the potential promise of the carbon polysulfide polymer as the cathode material for high performance lithium-sulfur battery.

## Introduction

With the rapid development of mobile electronic devices and electric vehicles on the market, the batteries to be used as power supplies are more urgently required to have higher performances. Therefore, the lithium-sulfur battery, which has a high capacity of 1675 mAh g^−1^ and a high energy density of 2600 Wh kg^−1^ in theory, is expected to be a promising next electrochemical energy storage system. In addition, sulfur is high natural abundance, environmentally friendly and low cost, which make it more desirable for commercial production^1–3^. However, the practical application of lithium-sulfur batteries is still hindered by their essential problems. For example, the sulfur and its discharge products are highly insulating and poor in reversibility, the large volumetric expansion of sulfur upon discharging results in electrode damage, and the high solubility of the intermediate products of polysulfides in the organic electrolyte leads to the so-called “shuttle effect”, namely, the parasitic reactions between the Li metal anode and dissolved polysulfides lead to poor battery performance. As a result, lithium-sulfur batteries show a relatively low active material utilization, poor cycling stability and low coulombic efficiency^4–11^.

A lot of strategies have been provided to address the problems mentioned above by physical or chemical confinement. These common methods are mainly focused on two aspects. On the one hand, in a pioneering work of Nazar as the representative, the sulfur is physically encapsulated with conductive materials to restrain the diffusion of polysulfides. Although these composites have shown improvement electrochemical performance while initial cycles, their electrochemical properties usually deteriorate in subsequent cycles because the individual physical confinement is not enough to trapping the polysulfides^12–21^. On the other hand, some chemical-confinement strategies have been proposed, such as fabricating doped carbon-sulfur composites and conductive polymer-sulfur composites or directly using elemental sulfur as a feedstock to form chemically stable copolymers, so as to improve the performance of the sulfur^22–32^. Owning to the strong chemical interaction between sulfur and the carbon framework in C-S copolymers, the dissolution and diffusion of polysulfides are effectively inhibited by chemical binding. Recently, Pyun and co-workers prepared a novel sulfur-rich polymer with 90 wt% sulfur content formed by the reaction of organic radical^30^. When fabricated by using this copolymer as cathode, the initial discharge capacity reached 1100 mAh g^−1^ at 0.1 C, however, fastly decayed to less than 400 mAh g^−1^ at 2 C. On the basis of this idea, Meng and co-workers prepared a sulfur-rich polymeric material through elemental sulfur and 1,3-diethynylbenzene copolymerization^31^. When fabricated by using this copolymer as cathode, which exhibited the initial specific capacity of 1143 mAh g^−1^ at 0.1 C and 595 mAh g^−1^ at 1 C. Although these sulfur copolymers provide a new way for the use of chemically confined polysulfides in the lithium-sulfur batteries, their poor conductivity has hindered the achievement of good cycling and rate performance. Li and co-workers designed a structure of carbon-sulfur matrix cathode with physical and chemical confinement, which was crucially important for high performance lithium-sulfur batteries. The sulfur-based cathode could be not only highly conductive to improve the utilization of sulfur, but also capable of effectively confining polysulfides to prevent their dissolution and accommodate the significant volume changes caused by lithium insertion/extraction^32^.

Here, we report a sulfur-rich carbon polysulfide polymer-graphene hybrid cathode for the lithium-sulfur batteries produced by chemical confinement strategies. This well-designed structure has several advantages: (1) the carbon polysulfide polymer has a highly uniform structure and high sulfur content; (2) the strong chemical interaction of sulfur with the carbon framework (C-S bonds) inhibits the shuttle effect; (3) the π electrons of the carbon polysulfide polymer enhance the transfer of electron and Li^+^; (4) the conductive graphene can provide the paths for fast electron transport and accommodate sulfur volume expansion. The combination of the physical characteristics of graphene and the strong chemical binding of C with S in the carbon polysulfide polymer enable high active material utilization during discharge/charge processes. A carbon polysulfide polymer-graphenen hybrid cathode delivers excellent cycling performance for 100 cycles with a high specific capacity of 600 mAh g^−1^ at the current density of 200 mA g^−1^.

## Results

### Synthesis and characterization of the carbon polysulfide polymer

**Figure 1:**
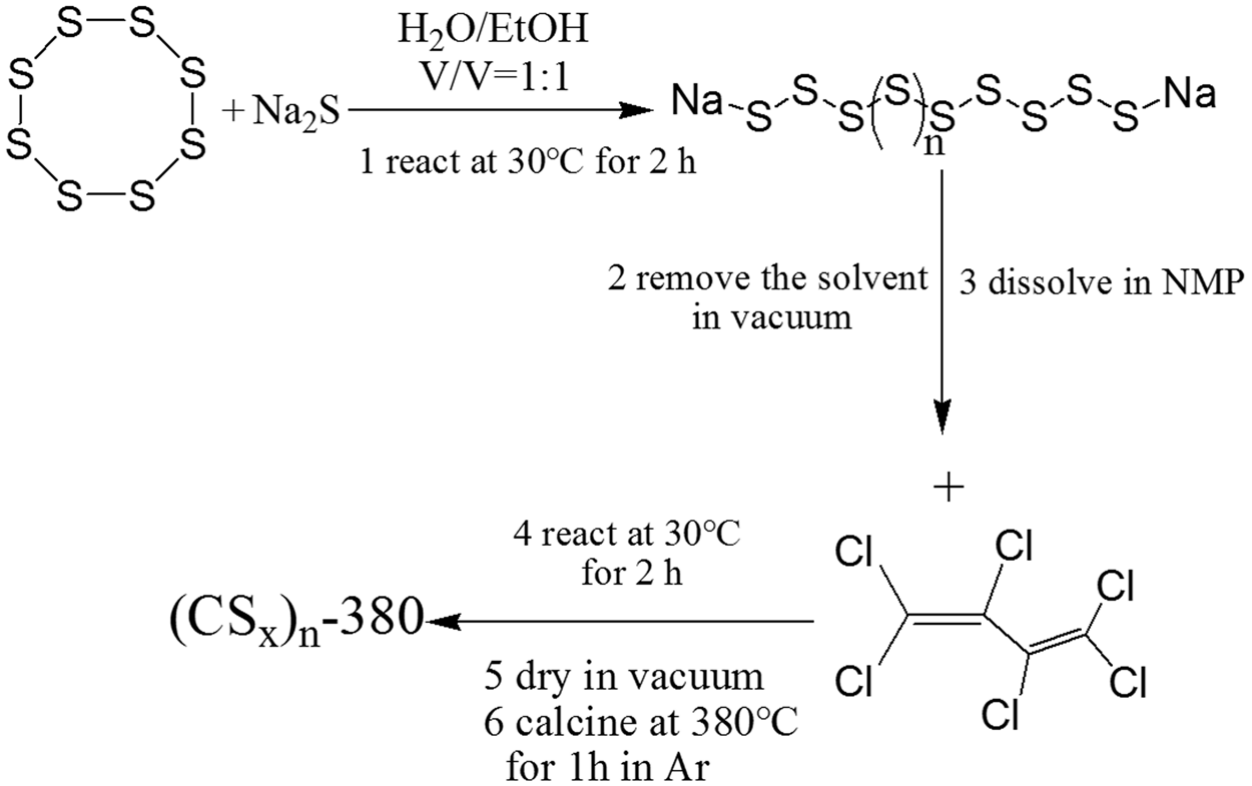
The preparation process of the carbon polysulfide polymer.

Figure 1 displays the preparation process of the carbon polysulfide polymer comprising carbon and sulfur as constitutive elements. Firstly, the polysulfides can be easily formed through the reaction of sublimate sulfur with sodium sulfide, which can be demonstrated by the color change of solution. As the reaction continues, the color of the solution will become dark gradually, indicating the gradually increasing chain length of the polysulfides (Figure S1, Supporting Information). Secondly, this process carried out by both the polymerization reaction of hexachlorobutadiene and the substitution reaction of polysulfide results in a chemically stable brown solid compound with a lot of polysulfide segments in the molecule (Figure S2, Supporting Information), which is marked as (CS_x_)_n_. Next, the above brown solid intermediate is heated under an argon atmosphere to evaporate impurities such as the polysulfide compound and the likely contained in the intermediate and to break the polysulfide segments in the molecule of the organic sulfur compound so as to evaporate and eliminate unnecessary sulfur. In order to determine the calcination temperature, we conduct thermogravimetric test on the (CS_x_)_n_ intermediate (Figure S3, Supporting Information). From the thermogravimetric analysis, we can see that weight loss of the intermediate is hardly observed above 380 °C. So the calcination temperature is set at 380 °C. Thus, a black gray carbon polysulfide polymer is obtained (Figure S2, Supporting Information) and marked as (CS_x_)_n_-380, which allows most of the carbon atoms in the molecule to bond to the sulfur atoms and also allows most of the sulfur atoms to form disulfide linkages having high reversibility in charging and discharging. To improve electronic conductivity of carbon polysulfide polymer, we add graphene dispersion liquid in the polysulfide synthesis process, the intermediate and final product are marked as (CS_x_)_n_-G and (CS_x_)_n_-G-380, respectively.

**Figure 2:**
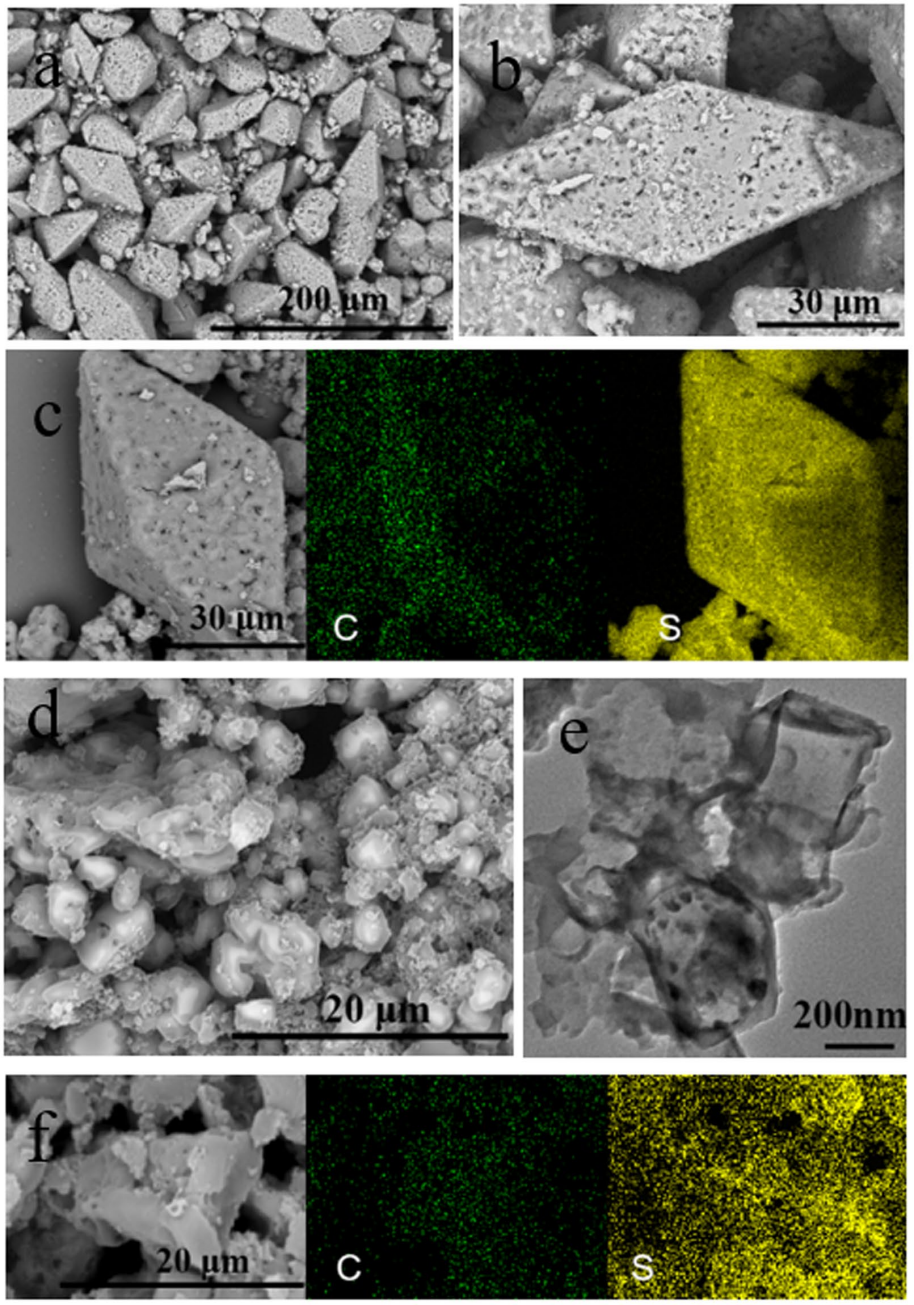
The morphologies of the obtained (CS_x_)_n_ and (CS_x_)_n_-380: (**a**,**b**) SEM images of the (CS_x_)_n_ intermediate, (**c**) element mapping of the (CS_x_)_n_ intermediate, (**d**) SEM image of the (CS_x_)_n_-380, (**e**) TEM image of the (CS_x_)_n_-380, (**f**) element mapping of the (CS_x_)_n_-380.

The morphologies of the obtained (CS_x_)_n_, (CS_x_)_n_-380, (CS_x_)_n_-G and (CS_x_)_n_-G-380 are characterized by scanning electron microscopy (SEM) and transmission electron microscopy (TEM). As we can see from the Figure 2a and b, the (CS_x_)_n_ intermediate is a porous prism with a few dozen microns. This porous structure is caused by the removal of sodium chloride from the reaction during the washing process. After calcinating at 380 °C, the intermediate is broken into irregular particles with size of microns (Figure 2d). TEM is further used to characterize the structure of the (CS_x_)_n_-380. As we can see from Figure 2e, the particles have a stable carbon structure which is helpful to improve the conductivity of active materials and accommodate sulfur volume expansion. Besides, in the (CS_x_)_n_-G intermediate, graphene disperses homogenously among the prismatic particles (Figure S4a and S4b), which is beneficial for the improvement of electronic conductivity of final product. After the calcination process, graphene is still evenly distributed in (CS_x_)_n_-G-380 hybrid which is helpful to improve the electrical conductivity of active materials (Figure S4d and S4e). To further ensure the distribution of the carbon and sulfur elements in the (CS_x_)_n_, (CS_x_)_n_-380, (CS_x_)_n_-G and (CS_x_)_n_-G-380, we test elemental mappings. The elemental mappings show the spatial distribution of sulfur and carbon, from the Figure 2c,f, S4c and S4f, we can see that the sulfur and carbon elements are homogeneously distributive in the (CS_x_)_n_, (CS_x_)_n_-380, (CS_x_)_n_-G and (CS_x_)_n_-G-380, indicating that the structure of the intermediate and carbon polysulfide polymer are uniform and the calcination process makes no difference in the microscopic distribution of the material elements.

The chemical structure of synthesized carbon polysulfide polymer is identified by Raman spectroscopy, Fourier transform infrared spectroscopy (FT-IR) and X-ray diffraction (XRD). As shown in Figure S5a, according to the result of the Raman analysis of the (CS_x_)_n_-380 carbon polysulfide polymer, there appears a main peak assigned to the carbon-carbon unsaturated bond (C = C bond) at 1444 cm^−1^, and only one peak appears at 505 cm^−1^ within the range of 400 to 525 cm^−1^. In other words, the carbon polysulfide polymer shows the peaks assigned to the disulfide linkages only but no peak assigned to the polysulfide segments. The Raman spectrum of the (CS_x_)_n_-G-380 composites is the same as that of the (CS_x_)_n_-380 and has no peak of graphene. This is because that graphene is wrapped in the particles of the (CS_x_)_n_-G-380 composites, which cannot be detected. FT-IR analysis is also performed to help understand the chemical structure of the carbon polysulfide polymer, and the results of FT-IR spectra are shown in Figure S5b. We can see that the peak positions of the (CS_x_)_n_-380 and (CS_x_)_n_-G-380 are the same, this indicates that the chemical structure of the carbon polysulfide polymer in the two materials is consistent. The absorption peak at around 1130 cm^−1^ is assigned as the C–S stretching, demonstrating that the C–S chemical bond forms through the preparation process^33–35^. The absorption peak at around 1630 cm^−1^ is assigned as the C=C stretching and the absorption peaks at around 467 cm^−1^ and 843 cm^−1^ are assigned as the S-S stretching. The result of FT-IR spectra is in agreement with the Raman spectra. The X-ray diffraction patterns of the (CS_x_)_n_-380, the (CS_x_)_n_-G-380 and the (CS_x_)_n_-9.5-380 are shown in Figure S6. All diffraction peaks of the (CS_x_)_n_-380 and (CS_x_)_n_-G-380 are in good agreement with the standard values of orthorhombic phase of sulfur (JCPSD no. 08-0247), indicating that the (CS_x_)_n_-380 and (CS_x_)_n_-G-380 contains long-chain elemental sulfur with the orthorhombic structure of elemental sulfur^31, 32^. For the carbon polysulfide polymer, the diffraction peaks of elemental sulfur are broad and weak. Particularly for the (CS_x_)_n_-9.5-380 carbon polysulfide polymer, the characteristic peaks of crystal sulfur are not observed, only a weak and broad diffraction peak at 25° remains and several diffraction peaks of the residual sodium chloride can be observed. The result demonstrates that all the elemental sulfur is polymerized.

### Electrochemical characterization

**Figure 3:**
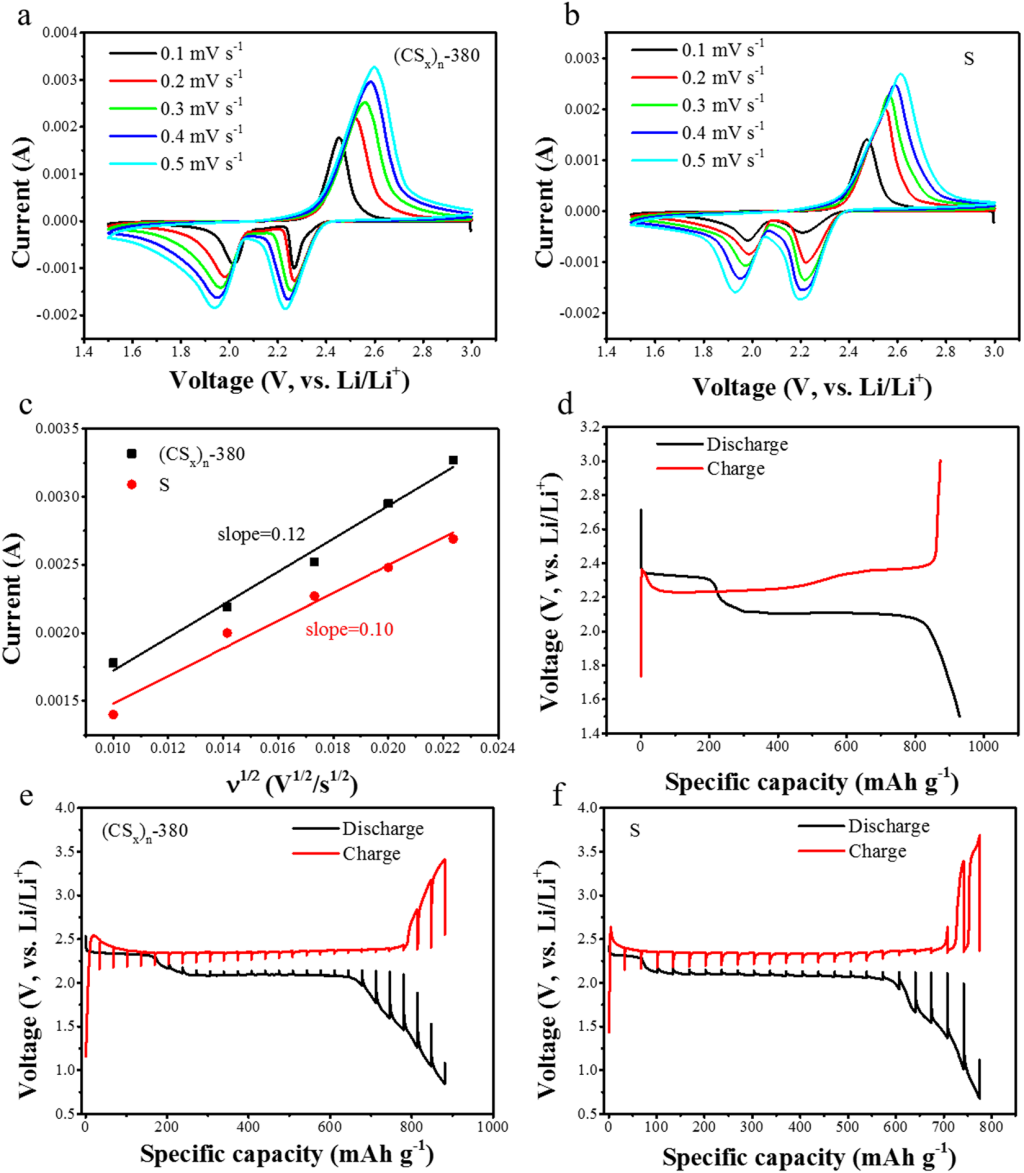
The electrochemical performance of the (CS_x_)_n_-380 as active material of the cathode: (**a**,**b**) the cyclic voltammetry (CV) of the (CS_x_)_n_-380 and S_8_ at different scan rates from 0.1 mV s^−1^ to 0.5 mV s^−1^ in the voltage range of 1.5~3.0 V versus Li/Li^+^, (**c**) corresponding i_pa_ − ν^1/2^ scatters and linear fitting of the (CS_x_)_n_-380 and S_8_, (**d**) the first charge-discharge curve of the (CS_x_)_n_-380 at the current of 200 mA g^−1^, (**e**,**f**) GITT curve of the (CS_x_)_n_-380 and S_8_ at 100 mA g^−1^.

The electrochemical performance of the (CS_x_)_n_-380 as active material of the cathode is investigated (Figure 3). The cyclic voltammetry (CV) data of the (CS_x_)_n_-380 and S_8_ are obtained at different scan rates from 0.1 mV s^−1^ to 0.5 mV s^−1^ in the voltage range of 1.5~3.0 V versus Li/Li^+^ and they are presented in Figure 3a and b. The two main reduction peaks for the (CS_x_)_n_-380 are similar with S_8_. The 2.3 V can be attributed to the reduction of sulfur to long-chain lithium polysulfide ions and the reduction of the carbon polysulfide to higher order organosulfur units. Continuing discharge into the lower voltage plateau results in the forming of Li_2_S_2_/Li_2_S and the conversion of oligosulfur units into fully discharged organosulfur products. In the anodic scan, a broad oxidation peak at 2.4 V is attributed to the conversion from short-chain to long-chain polysulfide, which is observed also in a few sulfur/conductive matrix materials, indicating a significantly improved reversibility, high conductivity and low polarization^32^. These analyses confirm that the carbon polysulfide (CS_x_)_n_-380 has similar electrochemical behavior with the S_8_. Figure 3d shows the first charge-discharge curve of the (CS_x_)_n_-380 at the current of 200 mA g^−1^, which comprises of two well-defined charge and discharge plateaus and is in agreement with the CV curves and similar to that of the S_8_. We can calculate the diffusion coefficient of Li^+^ according to the CV curves at different scanning rates based on the following Randles-Sevcik equation^36^:

1 $${{\rm{I}}}_{{\rm{p}}}=0.4463{\rm{nFAC}}{(\mathrm{nF}{\rm{\upsilon }}D/\mathrm{RT})}^{1/2}$$

where Ip is the peak current, D is Li^+^ diffusion coefficient (cm^2^ s^−1^),υis scan rate (V s^−1^), C represents concentration of Li^+^ (mol cm^3^), n is the number of electrons transferred, F is the Faraday’s constant (96485 C mol^−1^), A is electrode area (1.14 cm^2^). The C, n, F and A can be act as constant. Therefore, based on the equation 1, the value of D is proportional to the slope of dI_p_/dυ^1/2^. Chosen anode peak current in the CV curves to map υ^1/2^, the results are shown in Figure 3c. It can be seen that the slope of the (CS_x_)_n_-380 is greater than that of S_8_, indicating that Li^+^ diffusion coefficient of the (CS_x_)_n_-380 is greater than the S_8_. This is due to that the π electrons of the carbon polysulfide polymer enhance Li^+^ transfer. The galvanostatic intermittent titration technique (GITT) is used to analyze the dynamics of the (CS_x_)_n_-380. The results are shown in the Figure 3e and f. As we can see, the overpotential at the beginning of charge is relatively small, which indicates that, during the charge process of the (CS_x_)_n_-380, a short-chain polysulfide can more easily convert to long-chain polysulfide than S_8_, leading to a better reaction kinetics. The potential difference between the equilibrium potential and the maximum potential at the end of the current pulse is relatively smaller than the S_8_, which also indicates that there is a higher reaction kinetics in the (CS_x_)_n_-380.

## Discussion

Figure S7a shows the galvanostatic charge-discharge behavior of the (CS_x_)_n_-380 at the current of 200 mA g^−1^ in region of 1.5~3.0 V. From Figure S7a, we can see that the (CS_x_)_n_-380 delivers the capacity of ~700 mAh g^−1^. However, the capacity begins to decay after about ten cycles. We assume that it is not only because of the decomposition of LiNO_3_, but also because of the broken of the C-S bond in the (CS_x_)_n_-380 at the discharge cut-off voltage of 1.5 V so that the binding force between the polysulfides and the carbon backbone reduces. The shuttle of polysulfides and its reactions with the lithium result in the decreased utilization of active materials and the reduced capacity. To prove this assumption, we synthesize the (CS_x_)_n_-9.5-380 with more C-S bonds, which physical and chemical properties can be seen in Figure S8. The (CS_x_)_n_-9.5-380 is prepared as a cathode in the same method. When constant current charging-discharging at the current of 200 mA g^−1^ in region of 1.5~3.0 V, we obtain the charge and discharge curves shown in Figure S7b. It is observed that the first cycle discharge curve has a platform around 1.7 V, which may be attributed to the breakdown of C-S bond in fully discharged organosulfur products. The platform around 1.7 V does not exist at the second discharge, indicating that it is irreversible. That is a good proof of our assumption. In order to further prove that the platform around 1.7 V is due to the break of C-S bond, the (CS_x_)_n_-9.5-380 before and after 5 cycles in region of 1.5~3.0 V are characterized by X-ray photoelectron spectroscopy (XPS), the results are shown in Figure S9. It is observed that the peak at 284.7 eV is present in the (CS_x_)_n_-9.5-380 of before and after cycling, which is assigned as the C–S bond. However, after cycling, its peak area becomes smaller, indicating that the C-S bond is broken at the discharge cut-off voltage of 1.5 V. Thus we evaluate the subsequent electrochemical performances at the voltage interval of 1.8~2.6 V.

**Figure 4:**
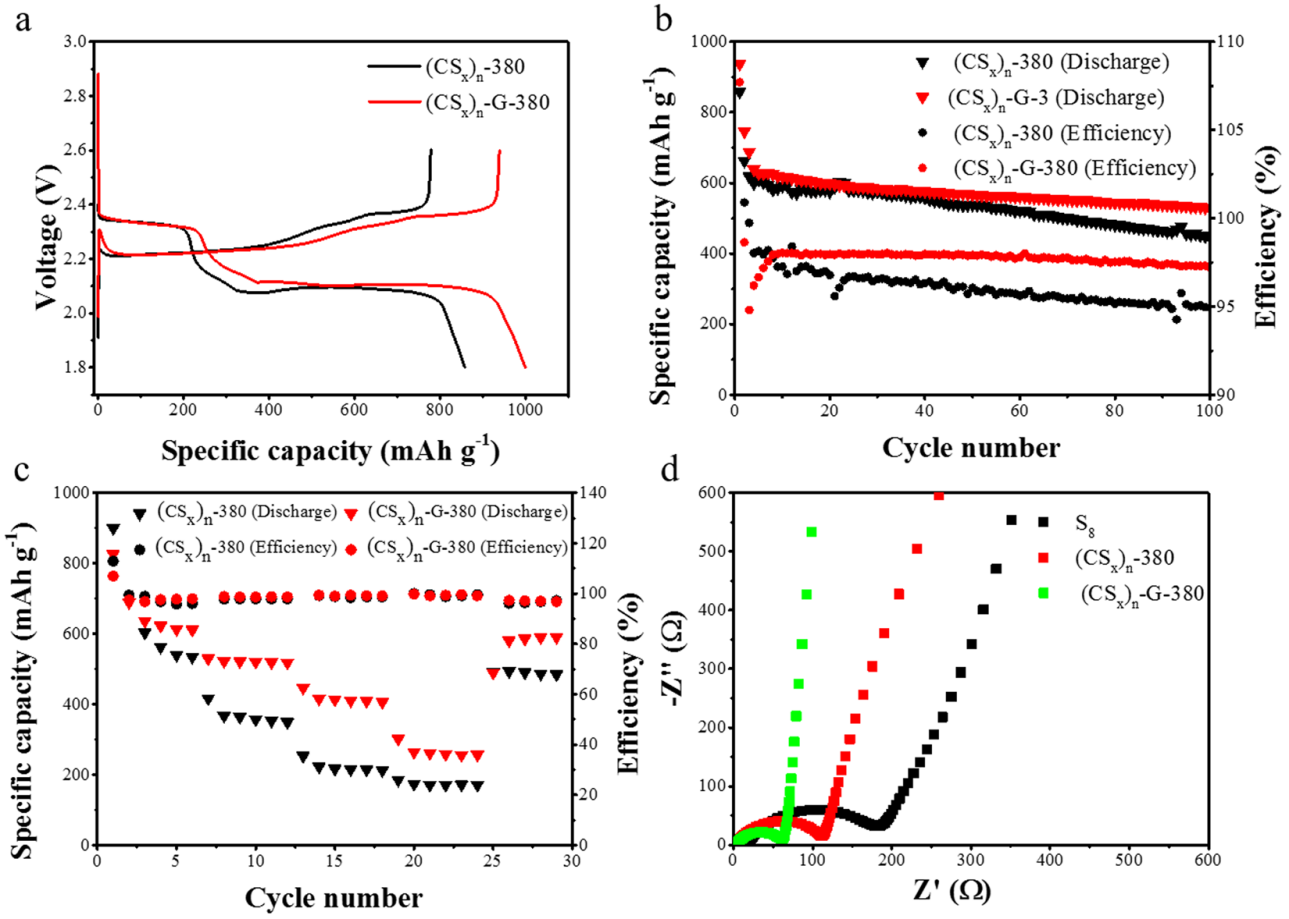
The electrochemical performance of the (CS_x_)_n_-G-380 and (CS_x_)_n_-380 as active material of the cathode: (**a**) the first charge-discharge curve at the current of 200 mA g^−1^ in region of 1.8~2.6 V, (**b**) the cycle performance at the current of 200 mA g^−1^ in region of 1.8~2.6 V, (**c**) the rate performance in region of 1.8~2.6 V, (**d**) the EIS.

The electrochemical performances of the (CS_x_)_n_-380 and the (CS_x_)_n_-G-380 when charge-discharge at the voltage of 1.8~2.6 V are shown in Figure 4. Figure 4a displays the initial charge–discharge curves of the (CS_x_)_n_-380 and the (CS_x_)_n_-G-380 at a current density of 200 mA g^−1^. During the charge-discharge, the charge-discharge curves consist of two well-defined charge and discharge plateaus, it turns out no change relative to the charge-discharge curve of S_8_. And the charge-discharge curves of both the (CS_x_)_n_-380 and the (CS_x_)_n_-G-380 show similar voltage plateaus during the charge/discharge processes. In spite of the similarities, we can see that there are still some obvious differences in the voltage hysteresis and specific capacity, which are correlated with the redox reaction kinetics and the reversibility of the battery. On the one hand, the voltage difference of the (CS_x_)_n_-G-380 battery between charge and discharge voltage platform is less than that of the (CS_x_)_n_-380, indicating a lower polarization and a kinetically efficient reaction process with a smaller barrier. On the other hand, the capacity of the (CS_x_)_n_-G-380 is 999 mAh g^−1^ (according to the mass of the (CS_x_)_n_-G-380, the same below), which is larger than that of the (CS_x_)_n_-380 (858 mAh g^−1^). These results demonstrate that the graphene in the (CS_x_)_n_-G-380 hybrid can increase the electrical conductivity of the cathode, resulting in lower polarization and thus improved utilization rate of active substances can be achieved. Figure 4b displays the long-term cycling performance and coulombic efficiency of the (CS_x_)_n_-380 and the (CS_x_)_n_-G-380 at a current density of 200 mA g^−1^. From Figure 4b, we can see that the cycle performance of the (CS_x_)_n_-380 has been significantly improved. An initial discharge capacity of 858 mAh g^−1^ based on the (CS_x_)_n_-380 mass is obtained. A very sharp capacity fade is observed over the initial five cycles and the capacity thereafter decreases slightly upon prolonged cycling, but it still has the discharge capacity of approximately 450 mAh g^−1^ after 100 cycles with a gradually declined coulombic efficiency. As added the graphene, the cycling stability, specific capacity and rate capability of the (CS_x_)_n_-G-380 are further improved. The discharge capacity is about 550 mAh g^−1^ after100 cycles with a relatively constant coulombic efficiency around 98%. Simultaneously, the (CS_x_)_n_-G-380 electrode has the good rate performance of 620 mAh g^−1^ at 200 mA g^−1^, 525 mAh g^−1^ at 500 mA g^−1^, 420 mAh g^−1^ at 1000 mA g^−1^ and 260 mAh g^−1^ at 2000 mA g^−1^, however, when charging and discharging at 1000 mA g^−1^, the (CS_x_)_n_-380 electrode has only the capacity of 210 mAh g^−1^. We have compared the performance of the current composite cathode with polymer based cathodes, polysulfide containing electrolyte and carbon/sulfur cathode for lithium-sulfur batteries, the results are shown in Table S1 ^30, 37–42^. As we can see from Table S1, the current composite material exhibits good electrochemical properties compared with similar polymer based cathodes. However, its electrochemical performance needs to be further improved compared with other polysulfide containing electrolyte and carbon/sulfur cathode.

In order to understand the excellent electrochemical properties of the (CS_x_)_n_-G-380, we have carried out the electrochemical impedance spectroscopy (EIS) test. As shown in Figure 4d, the EIS of all materials displays a depressed semicircle in the high frequency region and an inclined line in the low frequency region. The semicircle represents charge-transfer resistance R_ct_ of electrode/electrolyte interface. The semicircle diameter of the S_8_, the (CS_x_)_n_-380 and the (CS_x_)_n_-G-380 monotonically decrease, which indicates faster kinetics in the (CS_x_)_n_-G-380^43^. The R_ct_ of the (CS_x_)_n_-380 is smaller than that of the S_8_, which can be attributed to the π electrons of the carbon polysulfide compound enhancing the transfer of the electron and Li^+^. Moreover, the R_ct_ of the (CS_x_)_n_-G-380 is the smallest, this is because that the electrical conductivity of inner of the (CS_x_)_n_-G-380 particals is improved by the graphene. The results confirm that the (CS_x_)_n_-G-380 electrodes possess the high conductivity and remarkably enhance electron transfer during the charge and discharge. Therefore, the (CS_x_)_n_-G-380 electrode exhibits excellent electrochemical performance, namely superior cycling stability and high rate capability.

In summary, a novel high sulfur content carbon polysulfide polymeric material is prepared by a facile copolymerization of hexachlorobutadiene and substitution of polysulfide. The sulfur-rich carbon polysulfide ploymer shows a similar electrochemical activity with that of S_8_ but exhibits superb cycling stability and high coulombic efficiency. This is due to that the well-designed structure of the carbon polysulfide compound has several advantages, especially, the strong chemical interaction between sulfur and the carbon framework (C-S bonds) inhibits the shuttle effect and the π electrons of the carbon polysulfide compound enhance the electron and Li^+^ transfer. Furthermore, as-prepared carbon polysulfide polymer-graphene hybrid achieves outstanding cycle stability and relatively high capacity. This study highlights the potential promise of the carbon polysulfide compound as the cathode for high performance lithium-sulfur battery.

## Methods

### Preparation of carbon polysulfide sample

The first carbon polysulfide sample was prepared as follows: 25.0 g sodium sulfide nonahydrate (Na_2_S·9H_2_O) was dissolved in 75 mL water-ethanol mixed solvent (in a volume ratio of 1: 1), and 13.4 g sulfur was added subsequently. The mixture was reacted at 30 °C for 2 h and vacuum dried to remove the solvent afterwards, the residue was dissolved in 175 mL N-methyl-2-pyrrolidone (NMP) with 6.4 g hexachlorobutadiene added succedently. The mixture was reacted at 30 °C for 2 h. After that, the reaction mixture was vacuum filtered, thoroughly washed with deionized water, acetone and anhydrous ethanol, and dried in vacuum for 24 hours while holding the temperature at 60 °C. Thus, a brown compound was afforded as an intermediate, marked as (CS_x_)_n_. Next, 10 g the above intermediate (CS_x_)_n_ was put in a vessel of alumina, and the vessel with the (CS_x_)_n_ was placed on the center of the furnace. The (CS_x_)_n_ was heat-treated at 380 °C while flowing the argon gas and the programing furnace temperatures as the follows. That is, the furnace temperature was raised from room temperature to 60 °C in 0.5 h and kept for 1 h, and then raised to 380 °C in 2 h and kept for 1 h so as to eliminate a part of the sulfur atoms in the intermediate. Thus, the intermediate was converted to a carbon polysulfide, marked as (CS_x_)_n_-380. The sulfur content of the (CS_x_)_n_-380 reaches 90 wt%, as determined by the element analysis. The second carbon polysulfide sample was prepared according to the same method, the amount of hexachlorobutadiene was changed to 9.5 g and the prepared carbon polysulfide was labeled as (CS_x_)_n_-9.5-380. The third carbon polysulfide marked as (CS_x_)_n_-G-380 was prepared in the same way as well, the only difference is in the process of sodium polysulfide synthesis, 5 mL of 3.0 wt% graphene dispersion was added. The characterizations and clearly description of graphene are shown in Supporting Information.

### Materials Characterization

The morphology and structure of as-prepared materials were characterized by using a scanning electron microscope (SEM, HITACHI S-4800) equipped with energy dispersive X-ray spectroscopy (EDX) for elemental analysis and a transmission electron microscope (TEM, JEM-2100). The XRD patterns of the composites were recorded by the Philips X’pert Pro Super X-ray diffract meter and Cu Kα radiation. Element analysis was performed on a Vario EL III (Elementar Analysen Syetem GmbH, Germany) elemental analyzer. Thermogravimetric (TG) analysis was performed on a SDTQ600. The dried sample of 5~10 mg was placed in a Al_2_O_3_ pan and heated at 10 °C min^−1^ from 35 °C to 800 °C under a flow of nitrogen atmosphere. The laser Raman spectra were recorded at the resolution of 1 cm^−1^ in back scattering (180°) configuration using 532 nm excitation. X-ray photoelectron spectroscopy (XPS) was conducted on a PHI Quantum 2000 Scanning ESCA Microprobe.

### Electrochemical Property Measurements

The slurry with composition of 60 wt% carbon polysulfide active materials, 30 wt% super P and 10 wt% polyvinylidene fluorides (PVDF) dissolved in N-methyl pyrrolidone (NMP), was casted onto an aluminum foil and dried under vacuum at 60 °C overnight to prepare the working electrodes. The areal load of the electrode was about 0.8~1.0 mg cm^−2^. The CR2016-type coin cells were assembled using the prepared electrodes and lithium metal in an argon-filled glove box. The volume of the electrolyte in a coin cell was 50 μL. The electrolyte was formed by adding lithium bis(trifluoromethanesulfonyl)imide (LiTFSI, 1 M) salt and lithium nitrate (LiNO_3_, 1 wt%) into the mixture of 1,3-dioxolane (DOL) and 1,2-dimethoxyethane (DME) at the volume ratio of 1: 1. The galvanostatic charge-discharge experiments were measured at different current densities between 1.8 and 2.6 V or 1.5 V and 3.0 V (vs. Li^+^/Li) using a CT2001A cell test instrument (XINWEI Electronic Co.). In the voltage range between 1.5 V and 3.0 V (vs Li^+^/Li), at the scan rate from 0.1 mV s^−1^ to 0.5 mV s^−1^, the cyclic voltammogram (CV) curves were tested on a CHI660e electrochemical workstation. The electrochemical impedance spectroscopy (EIS) of the electrode was recorded by a Autolab PGSTAT 101 cell test instrument in the frequency range of 100 mHz~100 kHz, using two-electrode coin cells with Li metal as the counter electrode. All of the electrochemical tests were performed at room temperature. For GITT measurements, the cell was discharged and charged at a current density of 100 mA g^−1^ for 10 min, followed by open circuit relaxation for 10 min to reach equilibrium state.

## Electronic supplementary material

supporting information
