# Supplementary material for: High sulfur-containing carbon polysulfide polymer as a novel cathode material for lithium-sulfur battery
Source: Sci Rep. 2017 Sep 12;7:11386. doi: 10.1038/s41598-017-11922-6 (PMC5595856; doi:10.1038/s41598-017-11922-6)
Supplement: Supplementary file 1 — supporting information [file 41598_2017_11922_MOESM1_ESM.doc]

High sulfur-containing carbon polysulfide polymer as a novel cathode material for lithium-sulfur battery

*Yiyong Zhang* ^1^, *Yueying Peng* ^1^, *Yunhui Wang* ^1^, *Jiyang Li* ^1^, *He Li* ^1^, *Jing Zeng*, ^1^ *Jing Wang* ^1^, *Bing Joe Hwang* ^2^, *Jinbao Zhao* ^1^*

1 State Key Laboratory of Physical Chemistry of Solid Surfaces, State-Province Joint Engineering Laboratory of Power Source Technology for New Energy Vehicle, Collaborative Innovation Center of Chemistry for Energy Materials, College of Chemistry and Chemical Engineering, Xiamen University, Xiamen 361005, P. R. China

2 NanoElectrochemistry Laboratory, Department of Chemical Engineering, National Taiwan University of Science and Technology, Taipei 106, Taiwan

*Corresponding author. Tel.: +86-592-2186935;

Fax: +86-592-2186935

E-mail: [jbzhao@xmu.edu.cn](mailto:jbzhao@xmu.edu.cn)


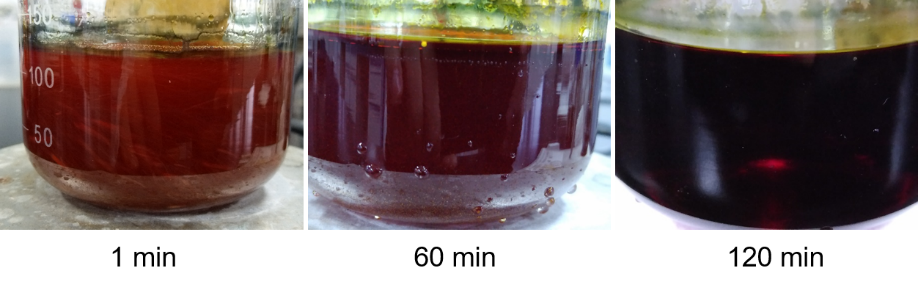


Figure S1. The color of the polysulfides solution as the reaction time increased.


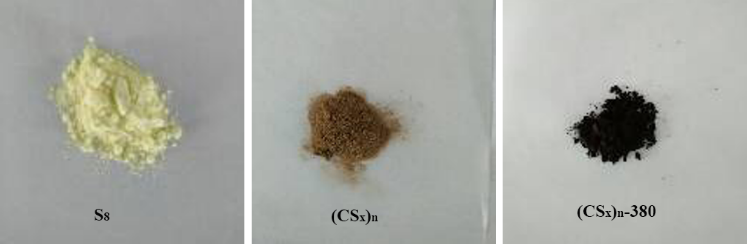


Figure S2. Apparent photographs of the S_8_, (CS_x_)_n_, and (CS_x_)_n_-380.


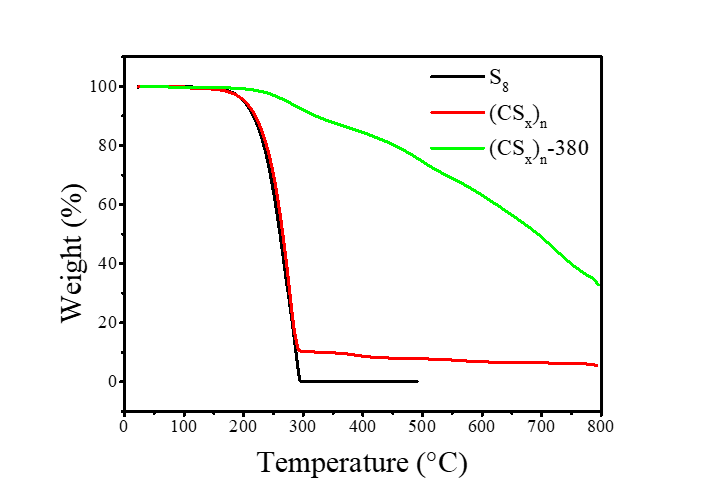


Figure S3. The thermogravimetric analysis of S, the (CS_x_)_n_ and the (CS_x_)_n_-380.


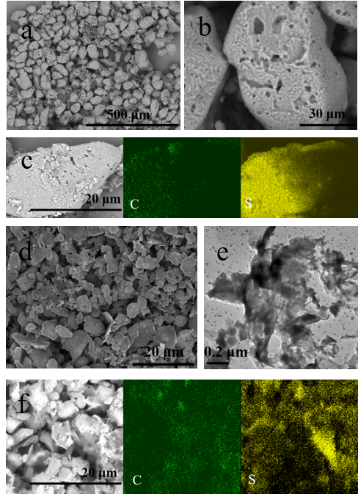


Figure S4. The morphologies of the obtained (CS_x_)_n_-G and (CS_x_)_n_-G-380: (a, b) SEM images of the (CS_x_)_n_-G intermediate, (c) element mapping of the (CS_x_)_n_-G intermediate, (d) SEM image of the (CS_x_)_n_-G-380, (e) TEM image of the (CS_x_)_n_-G-380, (f) element mapping of the (CS_x_)_n_-G-380.


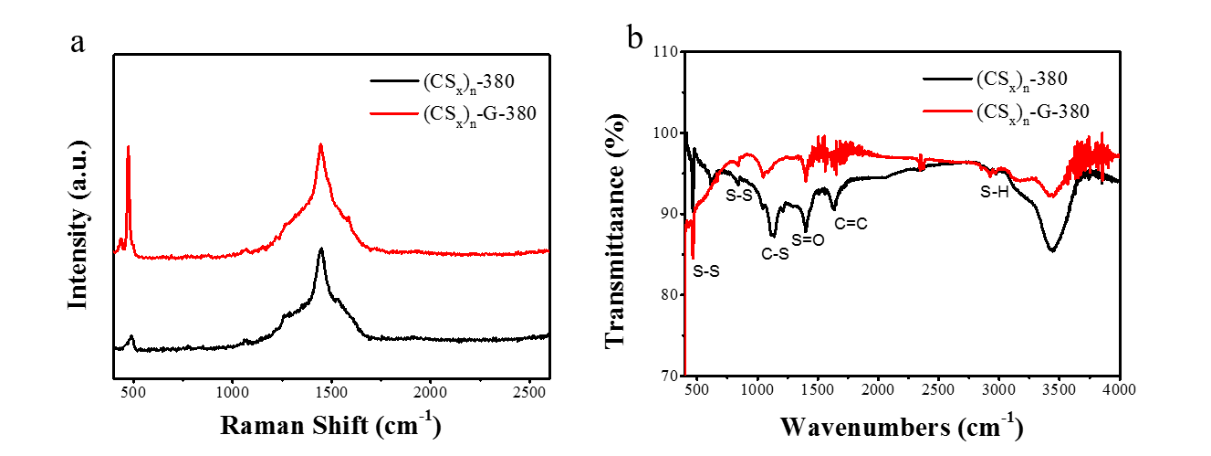


Figure S5. (a) the Raman spectra of the (CS_x_)_n_-380 and the (CS_x_)_n_-G-380, (b) the FT-IR spectra of the (CS_x_)_n_-380 and the (CS_x_)_n_-G-380.


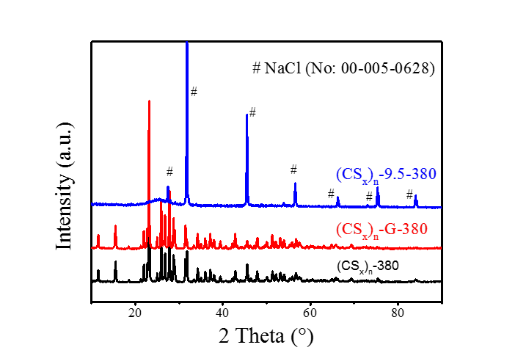


Figure S6. The X-ray diffraction patterns of the (CS_x_)_n_-380, the (CS_x_)_n_-G-380 and the (CS_x_)_n_-9.5-380.


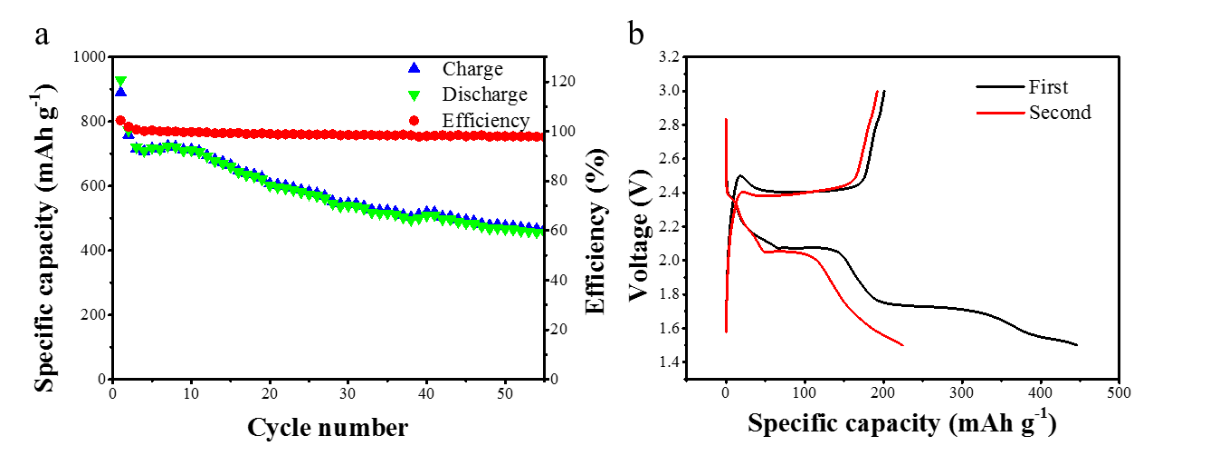


Figure S7. (a) the cycle performance of the (CS_x_)_n_-380 at the current of 200 mA g^-1^ in region of 1.5 ~ 3.0 V, (b) the charge and discharge curves of the (CS_x_)_n_-9.5-380 at the current of 200 mA g^-1^ in region of 1.5 ~ 3.0 V.


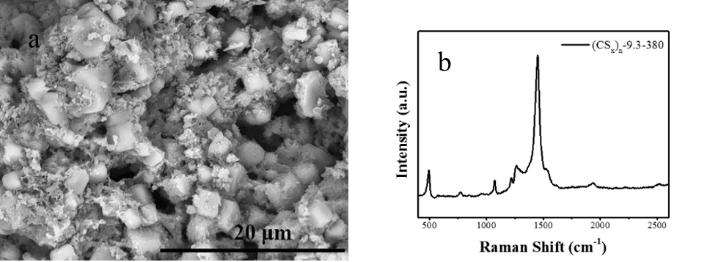


Figure S8. (a) SEM image of the (CS_x_)_n_-9.5-380, (b) Raman spectrum of the (CS_x_)_n_-9.5-380.


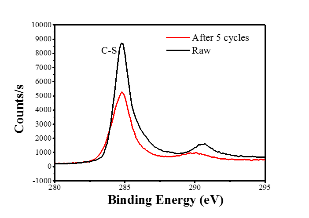


Figure S9. The XPS of the (CS_x_)_n_-9.5-380 before and after 5 cycles in region of 1.5 ~ 3.0 V.


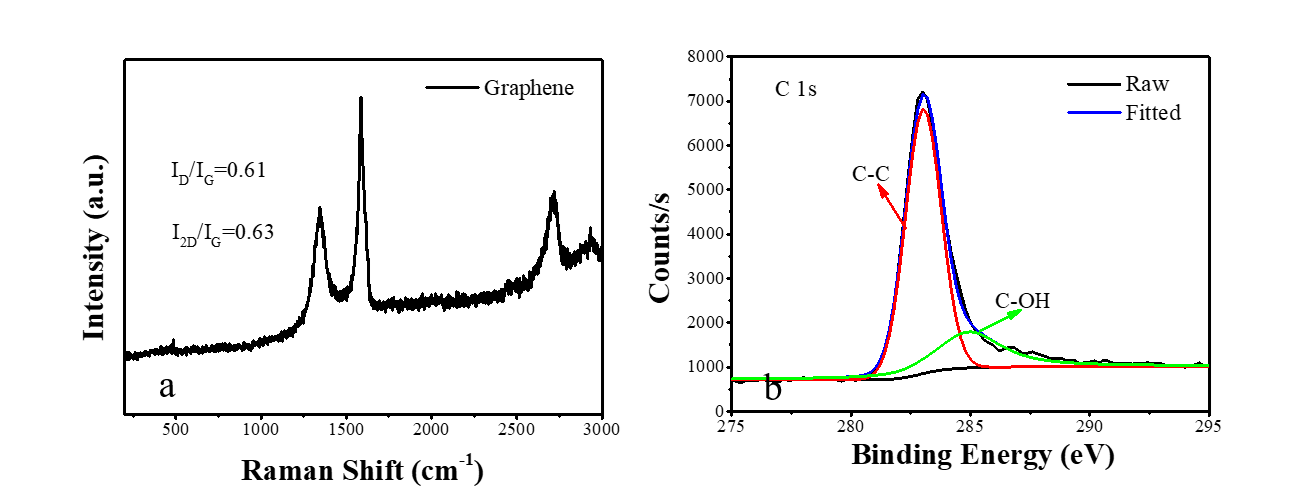


Figure S10. (a) Raman spectra of graphene sheet. The ratios of I_D_/I_G_ and I_2D_/I_G_ peaks are indicated in the figure; (b) XPS of the C 1s peak for graphene.

The deconvoluted XPS spectra of the C 1s peak (∼284 eV) show the presence of 5 at. % C-OH (285.4 eV) functional groups (Figure S10b). The Raman spectra of graphene sheet displayed an intense 2D and G peak at ∼2710 and ∼1586 cm^-1^, respectively (Figure S10a). Moreover, a defect-related D peak was observed at ∼1356 cm^-1^. The intensity ratio of D to G (i.e., I_D_/I_G_) was calculated to be 0.61. This is much lower than that of chemically or thermally reduced GO. The intensity ratio of 2D to G (i.e., I_2D_/I_G_) is normally related to the graphitization degree in graphitic carbons. The I_2D_/I_G_ ratio (0.63) of graphene sheets used in this work is significantly higher than that of rGO, further suggesting the high quality of the graphene. [8]

| **Composite** | **Mass**  **Loading (%)** | **Areal Loading (mg cm^-2^)** | **Composition (C/S: additive: binder)** | **Discharge current rate** | **Voltage range (V vs Li^+^/Li)** | **Initial Discharge capacity (mAh g^-1^)** | **After (nth) Discharge capacity (mAh g^-1^)** | **Ref.** |  |
| --- | --- | --- | --- | --- | --- | --- | --- | --- | --- |
| **Poly(S-r-DIB) copolymer** | **90** | **--** | **75:20:5** | **167 (mA g^-1^)** | **1.7 – 2.6** | **1100** | **823 (100)** | **1** |  |
| **PAN/S** | **53.4** | **--** | **7:2:1** | **0.2 mA cm^-2^** | **1.0 - 3.0** | **850** | **600 (50)** | **2** |  |
| **carbyne polysulfide** | **54.1** | **~ 2** | **7:2:1** | **168 (mA g_sulfur_^-1^)** | **1.0 - 3.0** | **~ 1400** | **~ 850 (200)** | **3** |  |
| **SMiP/SHEs** | **~ 55** | **5.7** | **--** | **1.5 (A g^-1^)** | **1.7-2.8** | **--** | **3.7 (mAh cm^-2^) (2000)** | **4** |  |
| **SD-C/S** | **81** | **1-2** | **85:5:10** | **0.2 (C)** | **1.8-2.8** | **1241** | **1042 (100)** | **5** |  |
| **CNT–S paper electrode** | **54** | **6.3** | **--** | **0.05 (C)** | **1.7-2.8** | **995** | **700 (150)** | **6** |  |
| **S-rGO-aerogel electrode** | **67** | **1.7-5.8** | **--** | **0.1 (C)** | **1.8-2.7** | **1000–1100** | **~ 500 (75)** | **7** |  |
| **(CSx)n-G-380** | **~ 90** | **~ 1** | **6:3:1** | **200 (mA g^-1^)** | **1.8 - 2.6** | **999** | **550 (100)** |  |  |

Table 1. Comparation on the performance of other cathode for Li-S batteries.

**References**

[1] Chung, W. J. et al. The use of elemental sulfur as an alternative feedstock for polymeric materials. *Nat. Chem.* **5**, 518-524 (2013).

[2] Wang, B. J., Yang, J. Xie, J., Xu, N. A Novel Conductive Polymer-Sulfur Composite Cathode Material for Rechargeable Lithium Batteries. *Advanced materials.* **14**, 963-965 (2002).

[3] Duan, B. et al. Carbyne polysulfide as a novel cathode material for lithium/sulfur batteries. *J Mater Chem A.* **1**, 13261-13267 (2013).

[4] Peng, H. et al. Healing High-Loading Sulfur Electrodes with Unprecedented Long

Cycling Life: Spatial Heterogeneity Control. *J Am Chem Soc.* **139**, 8458-8466 (2017).

[5] Ma, J. et al. Novel Large-Scale Synthesis of a C/S Nanocomposite with Mixed Conducting Networks through a Spray Drying Approach for Li–S Batteries. *Adv Energy Mater.* **5**, 1500046-1500052 (2015).

[6] Yuan, Z. et al. Hierarchical Free-Standing Carbon-Nanotube Paper Electrodes with Ultrahigh Sulfur-Loading for Lithium–Sulfur Batteries. *Adv Funct Mater.* **24**, 6105-6112 (2014).

[7] Nitze, F., Agostini, M., Lundin, F., Palmqvist, A. E., Matic, A. A binder-free sulfur/reduced graphene oxide aerogel as high performance electrode materials for lithium sulfur batteries. *Scientific Reports.* **6**, 39615-369622 (2016).

[8] Parvez, K. et al. Electrochemically Exfoliated Graphene as Solution-Processable, Highly Conductive Electrodes for Organic Electronics. *ACS Nano.* **7**, 3598-3606 (2013).
